# Epidemiological Aspects of the Initial Evolution of COVID-19 in Microregion of Uberlândia, Minas Gerais (MG), Brazil

**DOI:** 10.3390/ijerph18105245

**Published:** 2021-05-14

**Authors:** Deborah Araujo Policarpo, Eduarda Cristina Alves Lourenzatto, Talita Costa e Silva Brito, Daise Aparecida Rossi, Roberta Torres de Melo

**Affiliations:** 1 Laboratório de Epidemiologia Molecular, Faculdade de Medicina Veterinária, Universidade Federal de Uberlândia, Uberlândia 38400-000, Brazil; deborah.araujopolicarpo@hotmail.com; 2 Laboratório de Epidemiologia Molecular, Instituto de Biologia, Universidade Federal de Uberlândia, Uberlândia 38400-000, Brazil; eduardalourenzatto2009@gmail.com; 3 Superintendência Regional de Saúde, Setor de Vigilância em Saúde da Macrorregião de Uberlândia, Uberlândia 38400-000, Brazil; talitacostavet@yahoo.com.br; 4 Laboratório de Biotecnologia Animal e Aplicada, Faculdade de Medicina Veterinária, Universidade Federal de Uberlândia, Uberlândia 38400-000, Brazil; daise.rossi@ufu.br

**Keywords:** COVID-19, epidemiologic studies, incidence, multimorbidity, SARS-CoV-2

## Abstract

COVID-19 is considered by the World Health Organization to be a global public health emergency, which presents regional divergences that affect the epidemiological profile of the disease and are associated with political, economic, social and behavioral aspects. We aimed to analyze the epidemiological characteristics of the disease in the microregion of Uberlândia, Brazil, in order to determine risk factors that contributed to progression of SARS-CoV-2 virus. A cross-sectional study was conducted about micro- and macro-determinants combined with the significance analysis of suspected and confirmed cases in 18 municipalities during the epidemiological weeks (EW) 9 to 26. There were 34,046 notifications, of which 4935 (14.49%) people were diagnosed with COVID-19. Of these, 282 (5.71%) required hospital care and 40 (0.81%) died. Age and presence of associated comorbidities were decisive in the variations of incidence and lethality rates. In general, young people were the most affected and the elderly people, the most exposed to the serious and lethal form (*p* < 0.0001). Comorbidities such as diabetes and cardiopathies increased 33.5 times the death risk. The dispersion of the virus was centrifugal, in the inter as well as in the intra-municipal level. The disorderly implementation of municipal decrees applied in a decentralized manner in the municipalities seems to have contributed for the incidence rates increasing in the EW 25 and 26.

## 1. Introduction

The virus SARS-CoV-2 (Severe Acute Respiratory Syndrome Coronavirus 2) emerged the first time in China in December of 2019 and it is the cause of COVID-19 (Coronavirus Disease), considered by the World Health Organization (WHO) to be a global public health emergency [1]. Belonging to the family *Coronaviridae*, SARS-CoV-2 is an RNA virus that causes respiratory infections with a high rate of dissemination and zoonotic origin [2,3]. Worldwide, there are more than 838,000 deaths and 24 million confirmed cases of the disease. It possibly may be larger, since part of the population is asymptomatic [2,4,5]. In addition, the broad spectrum of symptoms and factors, such as population constitution, cultural, infrastructure and the decentralization of measures, affect the world in different ways, as well as hinder the fight against the disease [6].

In Latin America, the impacts are aggravated by problems related to sanitation infrastructure and cultural aspects related to the knowledge about epidemiological concepts such as social distancing and political issues [7,8,9]. In addition, in Brazil, the particularities in the social composition allied to comorbidities impact in different and significant ways on the number of cases and deaths [10]. Other factors that reinforce the regional individualities of the disease’s spread rate and consequently, the number of cases, are the Municipal Human Development Index (MHDI), ethnic, regional, city configurations, transport and social vulnerability [10,11,12,13].

In the pandemic scenario, local epidemiological monitoring plays a fundamental role to face the disease, since the analysis of the data allows the verification of specific patterns referring to COVID-19 in order to establish predictions of its behavior as well as its evolution. These patterns can present common characteristics in other regions and also at a global level, as observed in Portugal and Italy, which showed significant regional and local aggravations without national homogeneity in the cases [14,15]. It is known that the evident changes in the rigor of interventions carried out at the local level directly interfere with the transmissibility and the severity of the disease, as well as also generating different economic, social and behavioral consequences, as already reported in some countries and regions in Europe. Not only the type of social distancing applied in an unstable and/or imposing manner in different territories, but also personal behaviors, such as the simple habit of hand hygiene properly, also have a regional character [16,17,18]. In this way, local knowledge about the evolution of COVID-19 generates an important information for the rational direction of public policies in the sense of establishing a strategic plan for the effective control of the disease. 

With all national and regional singularities, it is crucial to monitor the progress of the disease and deepen understanding about the influence of comorbidities, adopted policies and demographic aspects to better characterize the epidemiology of the disease. In this work, we monitor the number of suspected and confirmed cases for the purpose of understanding the evolution of the disease related to political aspects and socioeconomic factors, in addition to the intrinsic risk factors of the populations affected in the microregion of Uberlândia at a specified period.

## 2. Materials and Methods

### 2.1. Structure

A cross-sectional study was conducted regarding suspected and confirmed patients for COVID-19 enrolled in the Regional Superintendence of Health (SRS) of the microregion of Uberlândia, Minas Gerais, Brazil. The data were analyzed in the initial phase of the pandemic from 25 February to 29 June 2020, corresponding to the defined epidemiological weeks (EW) 9 to 26. 

### 2.2. Definitions

The obtained data were grouped according to the classification of the patient as a suspect case, hospitalized suspect, confirmed and deceased. Suspected cases corresponded to those not tested for COVID-19, but reported as Severe Acute Respiratory Syndrome (SARS) and which had a negative result for the other etiological agents involved in respiratory syndromes (e.g., Influenza, Respiratory syncytial virus, Adenovirus, among others). The suspects hospitalized presented the same characteristics, however, associated with the need for hospitalization and/or intensive care. 

It was established as confirmed cases the group of people who presented positive results in laboratory test for RT-PCR COVID-19 (reverse transcription by polymerase chain reaction in real time) or by indirect rapid test for the detection of IgG and/or IgM as established by the Ministry of Health, Brazil [19]. 

### 2.3. Collection and Data Analysis

For data collection, we used the individual information obtained by the SRS of the microregion of Uberlândia, comprising the 18 municipalities included and classified as according to size related to the number of inhabitants (Table 1). 

The information used for the study included SARS notifications of suspected patients and those tested positive for COVID-19, in addition to demographic data, epidemiological and laboratory. All contents were subjected to filtration by double extraction method and organized at a time level according to the EW. 

For each group of municipalities, we determined the evolution of cases in a georeferenced way, the associated risk factors, and we have developed a critical analysis on the effects of decentralized government measures put in place. 

### 2.4. Statistical Analysis

Change Point Detection Algorithms by the At Most One Change (AMOC) method was used to identify points that occurred changes in the time series of suspected, confirmed, and death cases, by R software(version 3.6.3, R CoreTeam, Vienna, Austria) [21].

Continuous variables were shown as means and/or medians and were submitted to normality analysis by Shapiro–Wilk test or Kolmogorov–Smirnov test, followed by the application of Mann–Whitney test for analysis of two variables and Kruskal–Wallis test for three. Categorical variables were presented descriptively and we made comparisons between the suspected, confirmed cases, deaths, macro and micro factors, and categories of municipalities through Fisher’s exact test, as well as the identification of the main risk factors by determination of the odds ratio (OR). All tests were done by GraphPad Prism software (version 8.0.1, GraphPad Software, San Diego, CA, USA), considering statistical significance as a two-sided *p*-value < 0.05.

## 3. Results

**Figure 1:**
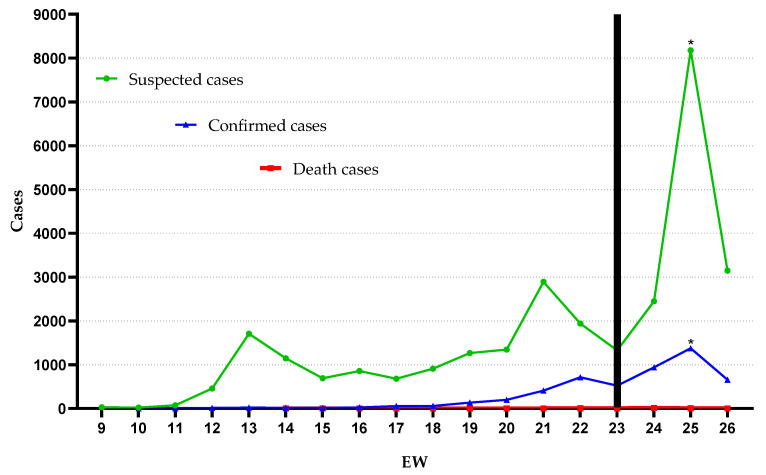
Suspected, confirmed, and death COVID-19 cases from epidemiologic weeks (EW) 9 through 26. Black bar: change point by AMOC. * *p* = 0.01, by Fisher’s exact test.

In total, 34,046 notifications were registered; 27,877 (81.88%) and 1234 (3.62%) of which treated suspect cases as not hospitalized and hospitalized, respectively. The median age for all suspected cases was 36 years (minimum 0 and maximum of 107 years). For suspected cases that required hospitalization, we found a median age of 59 years (minimum 0 and maximum of 104 years), being 637 (51.62%) men and 597 (48.38%) women. There were 4935 (14.49%) people who were diagnosed with COVID-19, and from these, 40 (0.81%) died. The algorithm detected only one change in temporal behavior, identified from EW 23 compatible with the rise of suspected and confirmed cases. In addition, we observed an increase in EW 25 that demonstrated a positive association in relation to the increase in the number of suspected cases (OR = 1.1; *p* = 0.01—Fisher’s exact test) (Figure 1). 

The median ages for confirmed cases and deaths was 38 (minimum 0 and maximum of 104 years) and 68 years (minimum 28 and maximum of 94 years), respectively. A frequency by gender was 49.40% (2438/4935) in women and 50.60% (2497/4935) in men, of whom 19 (47.50%) women and 21 (52.50%) men died. We observed that, under a general context, gender did not characterize a determining factor for the occurrence of suspected cases (*p* = 0.63), confirmed cases (*p* = 0.92) or deaths (*p* = 0.57; Mann–Whitney test). 

Individuals in adulthood, especially between 20 to 49 years, were the most affected by COVID-19 (*p* = 0.0002), but the number of deaths was significantly higher (*p* < 0.0001) in the elderly (26/40—65%) when compared to other age groups and the presence of comorbidities increased the risk of death by COVID-19 by 33.5 times (*p* < 0.0001; Fisher’s exact test) (Table 2). 

A total of 282 notifications required hospital care because they were associated with 11 general types of comorbidities present in the infected in a number from one to five distinct types present concomitantly, with a median of two comorbidities associated with each case of COVID-19. A total of 26/40 (65%) deaths linked to comorbidities were recorded, so that the existence of two or more comorbidities identified in 16/26 (61.53%) cases increased the risk of death by 2.85 (*p* = 0.02—Fisher’s exact test) times when compared to individuals who had only one comorbidity. For these lethal cases associated with multi-morbidities, cardiopathies and diabetes were the most relevant and present in 12/16 (75%) cases. It should be noted that the individual analysis of each comorbidity detected that the chance of death was higher in obese individuals (OR = 5.4), followed by patients with kidney diseases (OR = 2.57), cardiac patients (OR = 1.43) and patients with any lung disease (OR = 1.06). The percentages of cases and deaths related to each comorbidity are described in Table 3. 

The total number of infected per thousand inhabitants did not differ (*p* = 0.62) for small-sized (1.3 ± 0.6 cases/1000 inhab) or medium-sized (1.4 ± 0.8 cases/1000 inhab) municipalities, but for the cities that encompass to category of large-sized city, we detected 5.2 ± 1.4 cases/1000inhab (*p* < 0.0001) due to the previous inclusion of the disease in this group of municipalities. Despite the highest total percentage of infected women in small and medium-sizes municipalities, 56.7% and 51.3% respectively, gender was not a determining factor for the occurrence of COVID-19 considering the three categories of cities (small—*p* = 0.99; medium—*p* = 0.17; large—*p* = 0.12). The median age for cases of the disease also did not differ according to the size of the municipalities (Table 4). 

The involvement of macro determinants linked to HDI (human development index) [15] which are associated with income, schooling and longevity were not considered risk factors for the occurrence of cases in none of the municipalities’ categories investigated (*p* = 0.99). From monitoring the dispersion of the disease in the microregion of Uberlândia along the time, we observed that 17 of the 18 (94.44%) municipalities analyzed were affected by COVID-19 until EW 26, so that the greater dissemination between cities occurred in EW 20, in which we identified the inclusion of four municipalities of small and medium size. The centrifugal dispersion of the disease occurred in large cities, initially of EW 13 (minimum = 11; maximum = 13), for medium (median = EW 23; minimum = 17; maximum = 24) and small (median = 24; minimum = 20; maximum = 25) carriage. For large municipalities, it is evident that the occurrence of cases started in central or upper middle-class neighborhoods with a sense of dissemination to more peripheral locations, according to the georeferenced analysis of the evolution of cases (Supplementary Materials, Media S1). The later start of COVID-19 in small and medium-sized municipalities denoted densification of cases in municipal centers (S2 Media) and also in the central vicinity with isolated cases dispersed in the peripheries along the EW (S3 Media), respectively. Thus, the temporal analysis also justifies the higher occurrence of deaths in large municipalities (39/40—97.5%) and in central and/or upper middle-class neighborhoods (31/40—77.5%). 

**Figure 2:**
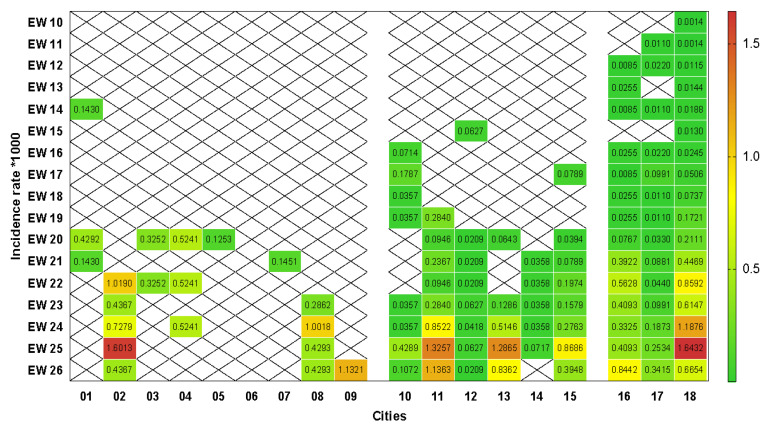
Heat graph based on extreme colors from green to red for rates of referring to incidence rates per COVID-19 per thousand inhabitants per epidemiological week in municipalities of small (01 to 09), medium (10 to 15) and large (16 to 18) size of the microregion of Uberlândia. EW: epidemiological weeks. X: absence of records. (Software: GraphPad Prism 8.0.1, San Diego, CA, USA).

The densitometric analysis of incidence rates is shown in Figure 2. We observed that small municipalities had a higher rate (median = 0.43; minimum = 0.13; maximum = 1.60) than medium (median = 0.08; minimum = 0.02; maximum = 1.33) and large (median = 0.05; minimum = 0.01; maximum = 1.64) size (*p* = 0.001; Kruskal–Wallis’ test). Despite the late insertion of the disease, smaller cities presented 3.5 times more chances of presenting incidence rates higher than 0.5 per thousand inhabitants than medium and large municipalities (*p* = 0.02; 95% CI = 1.1 to 2.6; Fisher’s exact test). 

The specific temporal analysis of each municipality showed that 50% of the cities had less than 0.5 cases per thousand inhabitants up to EW 20, the other 50% (01, 03, 05, 06, 07, 10, 12, 14 and 17) maintained incidence rates below 0.5 until the end of the study. The gradual increase in the incidence of the disease is more evident in the medium and large cities, so that 7/9 (77.77%) reached the peak of cases recorded in the study in EW 25, in addition to the small 02 municipality (Figure 2). In general, EWs 25 and 26 had incidence rates per thousand inhabitants significantly larger (*p* = 0.007; Kruskal–Wallis test) compared in relation to the other EWs. 

The discontinuity of cases over time was present in all categories of municipalities (13/17—76.47%) similarly (*p* = 0.81; Kruskal–Wallis test), so that three (3/13—23.07%) cities presented cases only in one epidemiological week, all of them from being all small, while 10/13 (76.92%) reported cases in alternate weeks (Figure 2).

## 4. Discussion

Recently, COVID-19 took place in China and spread worldwide, reaching Brazil in February 2020. The still fairly general knowledge related to the epidemiology of the disease restricts the complete determination of factors linked to the occurrence of the disease. In particular, the evaluation proposed by us at regional level has allowed a more realistic and approximate approach to the specific situation, considering important and specific aspects of the initial evolution of the disease. Based on this study, the proportion of severe versus common cases of SARS-CoV-2 infection in the first 17 weeks since the onset of the disease in the Uberlândia microregion was approximately 1:17. This value was much lower than that found in Beijing, whose authors identified a ratio of 1:4 [22] and close to the values found in Spain whose ratio was 1:15 [23], and in Sao Paulo was 1:25 [24]. It is known that COVID-19 affects people of all ages and backgrounds, but the differences found between studies show that some people are more likely than others to get sick and develop more severe forms of the disease if they are exposed to the virus, due to the presence of risk factors [25]. 

The micro-determinants of gender and age were of distinct relevance in this study (Table 2 and Table 4). Under none of the analytical perspectives performed do we observe the influence of gender on the occurrence or severity of COVID-19 cases, as observed in Beijing and in Oman [22,23,24,25,26] but different from countries such as China, Italy and the state of Louisiana in the United States, in which the first two countries found a relevant number of cases linked to the male gender, while in the state of Louisiana, female patients were the most affected [6,27]. In Brazil, in Macapá city, male patients received a greater number of notifications, whereas, in Pernambuco, confirmed cases were more frequent in women and deaths in men. The greater casuistry and severity identified in men described in some studies can be explained by the greater production of circulating antibodies in women and immunological factors linked to the X chromosome, as well as genes linked to innate and adaptive immunity, such as TRL7, involved in viral recognition [28,29,30]. Additionally and historically, men seek fewer health services, which indicates the existence of underreported cases, greater risk of worsening the disease, late treatment, and evolution to death [31]. The worldwide diversity of results related to the involvement or not of the gender factor can be explained by the magnitude of other genetic and non-genetic factors independent of gender that can alter susceptibility and mortality, including cultural habits in different countries [32]. 

The general population was susceptible to SARS-CoV-2, but young adults (20 to 49 years old) had a significant majority of confirmed cases (67.2%). The same age variation was also more prevalent in the cases of COVID-19 in the USA. In China, ages 30 to 54 had the highest number of cases [33,34]. This fact is due especially to the greater locomotion of this age group because of the imperative need to acquire resources through formal and informal work in person, which hinders the implementation of measures of social detachment. Both essential and non-essential services are composed mainly of young adults, aged 29 to 45 years, mainly [35,36,37]. Despite not representing the group with the highest lethality rate, young people have never been exempted from the risks associated with COVID-19, and as more young adults are infected, more and more of them may experience a debilitating disease with potential health effects in long-term. Greater seriousness must be considered for this group, not only to protect loved ones and communities but also for their own health [38].

The lethality rate in our study was 0.8%, but in the elderly, this value was 4.6%, which made clear the inclusion of this age group as a risk group for positive cases. Higher lethality rates in the elderly were also identified in a study conducted in Shanghai and the state of Rondônia in Brazil, with values equivalent to 10.6% and 13.2%, respectively [37,38,39]. Individuals aged 0–19 presented the lowest infection rates due to lower susceptibility to infection and/or no clinical symptoms of the disease [40]. This fact is explained by the differentiated immunity in this group, as among children, the innate response is high and the adaptive response is lower, which makes the response early, since T cells initiate the process of faster response. In addition, children have immature angiotensin-2 converting enzyme (ACE2), which decreases the affinity of SARS-Cov-2 virus and hinders the initial process of infection, while adults have mature ACE2 with higher levels of expression in the lungs, which may aggravate clinical signs [41,42]. The higher lethality in the elderly makes evident the greater demand for health care for this group. As policymakers make decisions about how to contain the pandemic and, at the same time, re-establish the economy, they must also be supported by population demographics and pay particular attention to the prevalence of older people in specific regions in order to determine areas with a particularly high risk of being affected [43,44].

The occurrence of deaths has shown to be higher when associated with the presence of comorbidities, such as diabetes, heart diseases and obesity. The association between disease severity and the presence of other comorbidities was also significant in a study conducted in Zhejiang, China, and also in Espírito Santo, Brazil [45,46]. The association of COVID-19 to cardiopathies generates systemic inflammation due to dysregulation linked to angiotensin-converting enzyme overexpression 2 (ACE2) and elevation of cardiac injury biomarkers [47,48]; in addition, the virus can cause myocardial dysfunction, which aggravates the clinical condition. Among the cases of diabetes, the increase in lethality is due to the difficulty of immune response, mainly innate, due to the hyperglycemic condition, the release of cytokines and the decrease in the release of interferon, responsible for antiviral function [49,50]. On the other hand, cases of obesity have a worsening in the immune response, due to systemic inflammation and consequently, impairment in the performance of defense cells, in addition to coagulation factors, which can lead to the formation of thrombi [51,52]. In addition, the association of two or more comorbidities having a greater impact on the risk of death and on the worsening of the clinical condition of COVID-19 has already been reported in the literature [46,47,48]. Thus, both the category and the number of comorbidities must be taken into account when predicting the prognosis in patients with COVID-19. In view of the severity related to cases associated with comorbidities, the priority inclusion of people from this group in the vaccination recommendations against COVID-19 becomes paramount as an appropriate measure to mitigate the number of fatalities.

The positive patients for COVID-19 were mainly residents of large cities (94%), since the evolution of the disease began in the most populous cities towards the medium and small municipalities. This same pattern of dispersion was identified in Wuhan, where the disease originated, with spread to Hubei provinces [53]. So, this fact was expected, and was identified in other locations in Brazil, and the first notifications were made in large centers, as São Paulo, Rio de Janeiro, Manaus, Belém, Fortaleza, Brasilia and metropolitan regions of Rio Grande do Norte, highlighting the fundamental role of air travel in the initial dispersion of the virus, which subsequently circulated by highways to the most peripheral locations [54,55]. Similarly, the microregion of Uberlândia met this standard, at inter- and intra-municipal level (Media S1, S2 and S3), since large cities have a high road flow, due to the strategic location connecting large centers in the country to peripheral regions [56]. This shows that the inclusion of sanitary barriers can represent a good strategy in breaking the chain of transmission from the flow of people in areas ranging from global to municipal scope. This finding is important for planning focused on the regions primarily affected or with the highest incidence of cases of COVID-19 [55]. 

Some studies demonstrated that the MHDI factor is interconnected to the occurrence of cases, with the number of deaths and confirmed cases being inversely proportional to the MHDI [57,58]. Although small, medium and large municipalities have different means of MHDI—0.7031, 0.7041 and 1.765 respectively, we did not detect interference of this macro determinant in the occurrence of cases, probably because the data are restricted to the initial stage of pandemic occurrence [20]. Early stages of the pandemic gained prominence for its denomination as ‘great equalizer’, capable of making anyone sick. However, with the further evolution and spread of the virus, reality shows that there are profound consequences linked to social inequalities in health, which make it evident that the main victims become economically vulnerable and without access to health care [59]. The fragility of this group makes it impossible to carry out measures related to the distance and isolation necessary to prevent transmission. In this sense, the early implementation of public financial, educational and health support policies for these people would function as a primary resource to control the future impact of the disease.

Considering the decentralized involvement of the legislative sphere in the population flexibilization and containment strategies in the period of our study, together with the mean COVID-19 incubation period of 14 days [60], we observed moments in which the incidence rates in some municipalities suffer oscillations according to the administrative measures adopted (Figure 2). In the municipality 18, we detected that the incidence rates assumed values higher than 0.5 from EW 22, concomitant to the opening of commerce considered non-essential, including academies, carried out by ordinance *n* 49,072 in EW 20, which reflected an isolation rate below 50% from that moment on. The control measures after this moment were restricted to the closing of malls and leisure areas and the reduction in the opening hours of commercial establishments [61], which did not prevent the occurrence of the highest incidence rate in this municipality (1.6 in the EW 25). 

The municipality 2 promoted the establishment of a municipal decree of containment (no. 3796) [62] only in EW 21, intended for the closure of non-essential activities and restaurants restricted to care via delivery. The late concern probably prevented the adhesion of the local population that registered high incidence rates (>0.5) in the following weeks (EW 22, 24 and 25). In parallel, municipality 11 presented peaks in the same EW, but the previous decrees (no. 5284, 5286 and 5295) were restricted only to the limitation of 50% of the capacity of the establishments, which justifies the increases. Already in the municipality 13, despite the implementation of Decree no. 029, since the EW 13, the stricter measures were only adopted with Decree no. 093 [63] in the EW 25, which included closure of non-essential services, mandatory use of masks in all establishments, individual passenger transport services and strengthening personal hygiene practices, which led to peaks in EW 25 and 26. Despite the implementation of Decree no. 84 in EW 18 by the municipality 15, whose measures returned to the distance and closure of non-essential services, from EW 19 there was an increasing easing through the reopening of bars and restaurants (Decree no. 92), operation of school activities in private institutions (extraordinary decision no. 11—EW 21), operation of driver training centers (extraordinary decision no. 12—EW 22), operation of foreign language schools, aesthetic academies and clinics (Decree no. 126—EW 23) [64]. Two weeks later, municipality 15 reached its peak (EW 25), demonstrating the coincidence with the incubation period of the disease. The other municipalities presented similar parameters or did not make the publication of the measures available. 

The disobedience to the distancing measures, the late implementation of containment decrees and the presence of unfounded flexibilization decrees showed compatibility with the rise of the disease identified from EW 23 by the change point algorithm in our study (Figure 1). This pattern was also identified in studies carried out in other metropolises and states in Brazil [65,66]. Especially in Belém, Brazil, a study emphasized that the influence of the economic needs of the municipality determined the disorganization in the control of the disease, which culminated in the overload of public health systems [67]. The lack of preparedness of the population, the need for financial resources and the unavailability of resources linked to individual protection against the disease, especially in vulnerable populations, are also relevant aspects for the advancement of the disease, but they could not be listed in our study [68,69]. 

Our findings suggest that future research should focus on the constant monitoring of micro and macro determinants of the disease in order to observe possible significant fluctuations in the later stages. At the same time, monitoring the effectiveness of the actions implemented is imperative at the local, regional, national and international levels, since the guarantee of people’s preventive behavior must be based on regulations consistent with the experienced situation. 

## 5. Limitations

Some limitations should be considered in this study. Variations in the number of suspected and confirmed cases, especially in EW 25 (Figure 1), which includes, in particular, the underreporting of cases, present not only in this study but also at the Brazilian and world level. In parallel, underreporting became even more evident due to the discontinuity of cases over time identified in all categories of municipalities demonstrated in our study (Figure 2). Reports of confirmed cases in the country are estimated to represent only 9.2% of actual results [70]. The estimate was also observed in Italy, the United States, and Spain, whose detection rate represents 1–2% of the real total of COVID-19 cases [27]. This determines the magnitude of the problem and its impact on the estimates presented in this study, mainly due to the difficulties of performing diagnostic tests, especially in the initial weeks used in our study. 

## 6. Conclusions

The epidemiological analysis of the initial moment of the COVID-19 pandemic in the Uberlândia microregion showed that age and presence of comorbidities were the most relevant factors in both the incidence and lethality rates of the disease. The centrifugal route of virus dissemination was maintained both inter- and intra-municipal. The late legal measures of containment, the early easing and the lack of compliance by the population showed coincidence in the gradual increase in the number of cases in the region. Special attention to risk groups and vulnerable populations and the implementation of health barriers represented possible mitigating measures for the problem. The specific epidemiological parameters demonstrated in our study are fundamental for a better understanding of disease patterns at the regional level and comparative to other regions and countries. The implementation of more effective strategies must be adopted and be in full agreement with the reality of each region, with surveillance in health and with the executive and legislative spheres. 

## Figures and Tables

**Table 1:** Municipalities in the Uberlândia Microregion, used in this study.

Municipality	Identifier	MHDI *	Population Density *	Size
Abadia dos Dourados	01	0.689	6989	S
Araporã	02	0.708	6869	S
Cascalho Rico	03	0.721	3075	S
Douradoquara	04	0.706	1908	S
Estrela do Sul	05	0.696	7978	S
Grupiara	06	0.731	1388	S
Indianópolis	07	0.674	6891	S
Iraí de Minas	08	0.695	6987	S
Romaria	09	0.708	3533	S
Subtotal (Mean)	-	(0.703)	45,618	-
Coromandel	10	0.708	27,974	M
Monte Alegre de Minas	11	0.674	21,120	M
Monte Carmelo	12	0.728	47,809	M
Nova Ponte	13	0.701	15,545	M
Prata	14	0.695	27,856	M
Tupaciguara	15	0.719	25,327	M
Subtotal (Mean)	-	(0.704)	165,631	-
Araguari	16	0.773	117,267	L
Patrocínio	17	0.729	90,757	L
Uberlândia	18	0.789	691,305	L
Subtotal (Mean)	-	(0.763)	899,329	-
Total (Mean)		(0.713)	1,110,578	-

MHDI = Municipal Human Development Index. S = Small-sized cities. M = Medium-sized cities. L = Large-sized cities. * Brazilian Institute of Geography and Statistics [20].

**Table 2:** Frequency and percentage of COVID-19 cases in the microregion of Uberlândia according to the age group.

Age Groups (Years)	Confirmed *n* (%)	Comorbidity *n* (%)	Deaths *n* (%)
<1	28 (0.56)	0	0
1–9	143 (2.89)	02 (0.70)	0
10–19	198 (4.01)	01 (0.35)	0
20–29	983 (19.91) *	13 (4.60)	01 (2.50)
30–39	1270 (25.73) *	19 (6.73)	06 (15.00)
40–49	1064 (21.56) *	41 (14.53)	04 (10.00)
50–59	682 (13.81)	58 (20.56)	03 (7.50)
≥60	567 (11.48)	148 (52.48) **	26 (65.00) **
Total	4935 (100)	282 (100)	40 (100)

* *p* = 0.0002; ** *p* < 0.0001 by Kruskal–Wallis test.

**Table 3:** Frequency and percentage of COVID-19 cases and deaths associated with comorbidities.

Comorbidities	Cases—*n* (%)	Deaths—*n* (%)
Cardiopathies	140 (49.64)	15 (57.69)
Diabetes	113 (40.07)	6 (23.08)
Pneumopathic	67 (23.75)	6 (23.08)
Kidney disease	20 (7.09)	4 (15.38)
Neurological disease	21 (7.44)	3 (11.54)
Obese	9 (3.19)	3 (11.54)
Hormonal disease	9 (3.19)	2 (7.69)
Neoplasia	8 (2.83)	2 (7.69)
Immunocompromised	15 (5.31)	0
Down syndrome	1 (0.35)	0
Liver disease	1 (0.35)	0

**Table 4:** Characteristics of those infected by COVID-19 by municipalities’ categories in the microregion of Uberlândia in EW 9 to 26.

Categories		Small	Medium	Large	*p*-Value	Total
Cases—*n* (%)		60 (1.22)	236 (4.78)	4639 (94.00) *	0.004	4935
Deaths—*n* (%)		-	01 (2.50)	39 (97.50) *	0.001	40
Median age (m, M)		44 (16, 89)	38 (0, 84)	38 (0, 104)	0.674	38 (0, 104)
Comorbidities *n* (%)	C	08 (2.84)	25 (8.86)	249 (88.30) *	0.002	282
	D	-	-	26 (100.00) *	0.001	26
Men *n* (%)	C	26 (1.04)	115 (4.61)	2356 (94.35) *	0.003	2497
	D	-	01 (4.77)	20 (95.23) *	0.009	21
Women *n* (%)	C	34 (1.40)	121 (4.96)	2283 (93.64) *	0.005	2438
	D	-	-	19 (100.00) *	0.001	19

m: minimum age, M: maximum age, C: cases, D: deaths, * *p* < 0.05 by Kruskal–Wallis test.

## Data Availability

The data used in this work are the same available in the website of Ministry of Health, available at https://www.gov.br/saude/pt-br and also through the plataform of the Electronic System of the Citizen Information Service (e-SIC), available at http://www.esic.cgu.gov.br/falabr.html.

## Supplementary Materials

The following are available online at https://www.mdpi.com/article/10.3390/ijerph18105245/s1, Media S1: Media in mp4 format, referring to confirmed cases in large municipalities during epidemiological weeks, Media S2: Media in mp4 format, referring to cases in small cities throughout the epidemiological weeks, Media S3: Media in mp4 format, referring to confirmed cases in medium-sized cities during epidemiological weeks.
